# Association of Cardiorespiratory Fitness with Achievement Motivation in Physical Education in Adolescents

**DOI:** 10.3390/ijerph18052317

**Published:** 2021-02-26

**Authors:** Cristina Cadenas-Sanchez, Javier Lamoneda, Francisco Javier Huertas-Delgado

**Affiliations:** 1 Institute for Innovation & Sustainable Development in Food Chain (IS-FOOD), Public University of Navarra, 31006 Pamplona, Spain; 2 PA-Help “Physical Activity for Health Promotion” Research Group, Junta de Andalucia, 11403 Jerez de la Frontera, Spain; educacionfisicajlp@gmail.com; 3 PA-Help “Physical Activity for Health Promotion” Research Group, La Inmaculada Teacher Training Centre, University of Granada, 18010 Granada, Spain; fjhuertas@ugr.es

**Keywords:** physical fitness, competence, music, youth, sex, shuttle run test, physical activity

## Abstract

Cardiorespiratory fitness is an important health marker in adolescents. Thus, examining the relation between cardiorespiratory fitness and motivation should be important to increase health-related behaviors. This study aimed to describe adolescents’ cardiorespiratory fitness and motivation by gender and to analyze the association between two cardiorespiratory fitness tests (original and with music) and motivation. A total of 341 adolescents (14.2 ± 1.5 years, 52.2% girls) participated in this study. Cardiorespiratory fitness was assessed using the 20 m shuttle run and its adaptation with music. Motivation was assessed though the “Achievement Motivation towards Physical Education” questionnaire. Boys presented with higher cardiorespiratory fitness and motivation (all, *p* < 0.05). Yet, when classifying fit and unfit groups, a higher percentage of girls were considered fit compared to boys (85.8% vs. 74.5%). A higher level of cardiorespiratory fitness (stages) and VO_2_max were associated with a higher level of motivation (self-perceived competence and compared competence) and lower anxiety (all *p* < 0.05). These associations with motivation were stronger when the music was present in the test. In this sense, including music in activities focused on cardiorespiratory fitness could increase the cardiorespiratory fitness performance and motivation, especially in girls. It should be important to increase adolescents’ cardiorespiratory fitness levels in order to increase motivation in physical education lessons and to include more motivational activities in order to achieve higher performance.

## 1. Introduction

High amounts of sedentary behavior have been considered as an important risk factor of chronic conditions in the 21st century [1]. Compelling evidence suggest that increasing physical activity act as a polypill to counteract all the health-related consequences derived from sedentarism in children and adolescents [2]. The new launched global guidelines on physical activity and sedentary behavior provided evidence that greater amounts and higher intensities of physical activity, as well as different types of physical activity (aerobic and muscular activities) were associated with improved health outcomes. In order to achieve these benefits, youth should engage in an average of 60 min of moderate-to-vigorous physical activity daily, and increased time in aerobic activities could lead to incremental benefits [3]. Linked to physical activity, physical fitness refers to a person’s ability to perform physical activities, and is considered a powerful marker of health [4]. Noteworthy, a recent scientific statement by the American Heart Association concluded that healthy cardiorespiratory fitness is positively associated with cardiovascular health, as well as brain health in youth [4]. Furthermore, it is important to assess and increase cardiorespiratory fitness levels, as it can be tracked from the early ages to the elderly [5].

The assessment of cardiorespiratory fitness is proposed to be used in preventive health [6]. In this sense, schools are an ideal setting to promote and assess health-related behavior [7], as they provide the best way to target the entire population, as virtually all children attend school [8]. The most-used test in the school context is the 20 m shuttle run test (hereinafter, the “SRT-original”), developed by Léger et al. in 1988 [9], with published data on over 1 million children from 50 countries [10]. Recently, we released a feasible and valid adaptation of this test (named as the 20 m shuttle run with music (SRT-music)) in order to increase motivation and levels of enjoyment in the adolescents using music throughout the test [11].

A meta-analysis conducted on motivation and healthy behaviors showed that motivation is an important determinant of physical activity [12]. It is especially important in adolescence, as it is presented as a period when both physical activity and motivation levels decline [13], even during physical education lessons [14]. In order to increase physical activity levels in adolescents, motivation towards physical activity should at least be maintained [15], especially during physical education lessons, as motivation has been shown to be a key to increase physical activity and health-related behaviors [16]. The achievement motivation theory presents perceived competence as the antecedent of achievement goals [17]. Moreover, achievement goal motivation has been related with better experiential and performance outcomes in sports participants [18]. In this sense, psychological needs referred to by the self-determination theory (i.e., competence, autonomy and relatedness) are crucial to enhanced self-motivation and also mental health [19]. Specifically, the self-perception of individual abilities and competence are associated to physical activity during adolescence and related to later physical activity behavior [20]. Thus, achievement goal motivation (hereinafter, motivation) is important to determine behavior in physical education settings, as well as their performance and participation [21]. Consequently, the inclusion of music in sports has been related with higher levels of motivation and positive affect [22]. Furthermore, the inclusion of music in physical activity exercises can increase enjoyment and even performance [23]. Nevertheless, music helps adolescents to satisfy their emotional needs [24], and the enjoyment has been shown to be related to the autonomous motivation, as well as the self-perception of their own abilities [25]. In addition, the inclusion of a music fitness program during physical education has been proved to be effective to increase physical fitness [26]. However, according to our knowledge, music has not been used before to increase motivation during fitness assessment in physical education, and less is known about the relationship between cardiorespiratory fitness and motivation.

Therefore, the main purposes of this work were: (1) To describe the cardiorespiratory fitness and motivation levels of Spanish adolescents by gender, and (2) to analyze the association between cardiorespiratory fitness (assessed with the SRT and the SRT-music tests) and motivation in adolescents.

## 2. Materials and Methods

### 2.1. Study Design and Participants

Out of 505 participants that were initially enrolled in the project, a total of 341 students (52.2% girls; age ranged from 12 to 18 years, body mass index ranged from 14.8 to 40.3 kg/m^2^) with valid data on at least one cardiorespiratory fitness test and motivation levels were included in this study (*n* = 164 excluded for not having completed data because they did not attend school). No differences were found regarding age, sex, body mass index, cardiorespiratory fitness, and motivation levels between the current study sample and the rest of the project sample (all *p* > 0.05). Students were selected by non-probability sampling from two different schools in Cadiz (south of Spain). Data collection took place from May 2018 to June 2019. Data collection was performed by the same evaluator, yet the person who conducted the analyses was blinded to exposures and outcomes, and was not part of the data collection. This cross-sectional study is part of the “Adaptation of cardio-respiratory capacity assessment instruments for the improvement of motivation, perception of effort and physical performance in adolescents” project, funded by the Regional Ministry of Education of Andalusia, Spain (PIV-009/18).

The study protocol was approved by the research committee of the Regional Ministry of Education of Andalusia and by the school boards following the principles of the Declaration of Helsinki. All the parents or legal guardians of the students signed an informed consent form.

### 2.2. Measures

#### 2.2.1. Anthropometric Measures

Weight was measured with a scale (precision 0.1 kg; Decathlon, Villeneuve d’Ascqcedex, France). Height was measured in the Frankfort plane, using a tape measure (precision 0.1 cm; Elk Sport, Zaragoza, Spain). The weight (kg) and height (cm) were measured once without shoes and in light clothing (underwear) by an expert in physical activity and health. Body mass index (BMI) was therefore calculated (weight in kg divided by height in cm squared).

#### 2.2.2. Cardiorespiratory Fitness: The 20 m SRT-Original and the 20 m SRT-Music

Cardiorespiratory fitness was evaluated twofold, by the 20 m shuttle run test in its original version (SRT-original), and a recently validated adaptation with music, named the 20 m shuttle run test with music (SRT-music) [11]. The tests consisted of running back and forth for a distance of 20 m following an audio signal. The tests started at 8.5 km/h and increased every minute by 0.5 km/h. The tests finished when the participant stopped because of exhaustion or when they did not reach the end lines concurrent with the audio signal on two consecutive occasions. These tests were performed only once, and the number of laps were registered and used for the analyses. The maximum oxygen intake (VO_2_max) was therefore estimated by an equation (i.e., Y = 31.025 + 3.238 X − 3.248A + 0.1536AX, where X refers to maximal speed reached and A to the age) [9]. Additionally, we recorded the heart rate during the tests using heart rate monitors (ONrhythm 500, Decathlon, Villeneuve d’Ascqcedex, France), and the maximum heart rate achieved at the end of the tests was recorded.

The unique difference between the original 20 m shuttle run and its adaptation was the inclusion of audio tracks (i.e., music) into the original file. This adaptation has been shown to be feasible and valid in comparison with the original test [11]. The tests were equally counterbalanced per classroom within a two-week period between measurements. Detailed information about the adaptation of the 20 m shuttle run with music can be found elsewhere [11].

#### 2.2.3. Motivation

Achievement motivation in physical education was assessed by the Spanish version of the Achievement Motivation in Physical Education questionnaire [27]. The questionnaire consisted of 32 items comprising four subscales: (i) Perception of self-perceived motor competence (e.g., “So far, I am good at physical education without really trying hard”); (ii) perception of comparative motor competence (e.g., “I have always considered myself to be the best in physical education”); (iii) commitment to learning (e.g., “I usually listen to the things my physical education teacher tells me”); and (iv) general anxiety and anxiety in the face of failure (e.g., “I often get nervous when practicing in public”) [27]. The responses were ranged from strongly disagree to totally agree (from a 1 to 5 Likert scale). Furthermore, the reliability of the questionnaire and the subscales was considered acceptable (Cronbach’s alpha ranges from 0.778 to 0.909). The questionnaire was performed before the cardiorespiratory fitness tests in order not to influence their performance to their responses.

### 2.3. Statistical Analyses

Descriptive characteristics of the entire sample and by gender are presented as mean and standard deviations or frequencies. The Student’s t-test was used for examining differences between boys and girls in the descriptive characteristics. Further, we explored whether gender and age could influence the results by checking interaction analyses, and the results remained similar (data not shown). Therefore, the data presented in this study compile both genders.

In order to examine the association between cardiorespiratory fitness and motivation, linear regression analyses adjusted for age, gender, and body mass index were applied. Cardiorespiratory fitness assessed by both the SRT-original and the SRT-music tests was included as an independent variable. Motivation components (i.e., self-perceived competence, compared competence, commitment, and anxiety) were included as dependent variables, and all the analyses were adjusted for age, gender, and body mass index.

Additionally, to test whether those adolescents grouped as fit presented with lower or higher motivation, analysis of covariance (ANCOVA) adjusted for age, gender, and body mass was applied. Fit and unfit categorization for each test was made based on the published health-related cut-off points (fitness levels below 42 and 35 mL/kg/min for boys and girls, respectively) [28].

All the statistical procedures were performed using the SPSS software for Windows (version 22.0, IBM Corporation, NY, USA). A significance level of *p* < 0.05 was set for the analyses.

## 3. Results

### 3.1. Descriptive Characteristics

Table 1 shows the descriptive characteristics of the study sample (all, boys and girls). Briefly, differences between boys and girls were found in cardiorespiratory fitness (both SRT-original and SRT-music) and motivation levels (i.e., self-perceived competence, compared competence, and anxiety) (all *p* ≤ 0.011). Further, we analysed the differences between the SRT-original and SRT-music tests. The results showed significant differences in cardiorespiratory fitness measured by stages and VO_2_max, all in favour of SRT-music (all *p* ≥ 0.012) (data not shown).

### 3.2. Associations Between Cardiorespiratory Fitness and Motivation

Table 2 shows the associations between cardiorespiratory fitness and motivation levels. In brief, the results showed that those adolescents with higher performance in both cardiorespiratory fitness tests showed greater self-perceived competence (all β ≥ 0.36, *p* < 0.001), compared competence (all β ≥ 0.34, *p* < 0.001), commitment (all β ≥ 0.18, *p* < 0.05), and lower anxiety (all β ≥ −0.34, *p* < 0.001). Overall, higher standardized beta coefficients were found in the 20 m SRT-music test compared to the 20 m SRT-original.

**Figure 1:**
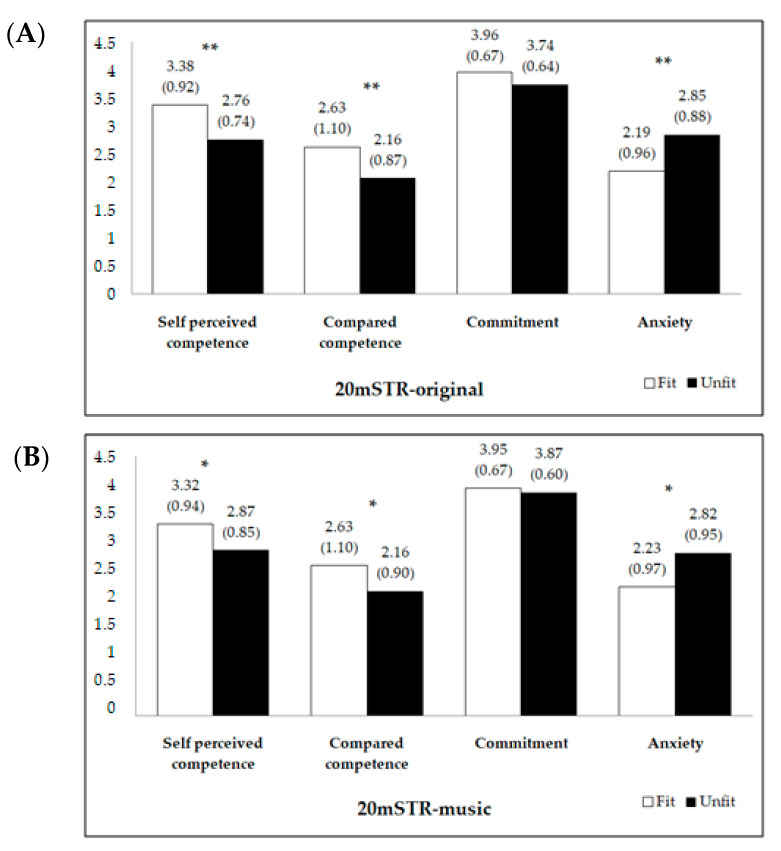
Motivation levels between fit and unfit adolescents using the original 20 m shuttle run test (20mSRT-original, panel (**A**)) and the adapted 20 m shuttle run test with music (20mSRT-music, panel (**B**)). The difference between fitness groups (fit vs. unfit) was calculated using analysis of covariance, adjusting for age, gender, and body mass index. * *p* <0.05 ** *p* < 0.001.

Further, Figure 1 compares the motivation levels between those adolescents who were grouped as fit to those peers who were grouped as unfit. We observed that for both cardiorespiratory fitness tests, those adolescents who were fit presented greater levels of motivation and lower levels of anxiety than those peers who were unfit (Figure 1, all *p* ≤ 0.026). The differences between fit and unfit groups were lower in the 20 m SRT-music test compared to the original one.

## 4. Discussion

This study aimed to describe the cardiorespiratory fitness and motivation levels of a sample of Spanish adolescents by gender, and to examine the association between cardiorespiratory fitness assessed by the SRT-original and SRT-music tests, and motivation levels. The main results of this study were: (1) Boys reported higher levels of cardiorespiratory fitness and motivation than girls, and (2) higher levels of cardiorespiratory fitness were associated with higher motivation (self-perceived and related competence) and lower anxiety. Moreover, the fit group reported higher motivation levels than those peers classified in the unfit group. Further, these associations were stronger in the test with music compared to the original test.

Our findings show that boys reported higher levels of cardiorespiratory fitness, and higher motivation and lower anxiety than girls. According to previous research, boys presented higher levels of cardiorespiratory fitness than girls, which in turn could be related to their biological maturation process [30]. In addition, the physical activity level has been associated to a higher fitness level [31,32], and boys tend to present with higher levels of physical activity than girls [33]. These differences could be related to societal gender inequality, as this has been associated with larger differences between boys and girls [34]. However, even when cardiorespiratory fitness levels were higher for boys, a higher percentage of girls who accomplished the fitness level were considered fit when the test was conducted with music. Tomkinson et al. presented similar results in their systematic review compelling nearly 3 billion children and adolescents [35]. Music has been previously associated with higher performance in girls but not in boys [36], and it could explain the higher differences found in our study between genders in the SRT-music test. Moreover, motivation towards physical education was also higher in boys than in girls. Therefore, it is important to deepen the curricular and motivational aspects during physical education, as it has been evidenced that physical education programs are not effectively meeting girls’ needs [37]. In addition, promoting the use of different teaching styles (e.g., gamification), which is related to higher levels of motivation in adolescents with regard to physical education, is a good strategy to reduce gender inequality in motivation levels [16].

A higher level of cardiorespiratory fitness was associated with higher motivation towards physical education. Cardiorespiratory fitness has been strongly associated with other psychological outcomes [38,39]. Specifically, Ishinara et al. reported specific associations between physical fitness and motivation via exercise habits in children, but not a direct association between physical fitness and motivation [40]. In addition, physical fitness was positively related to perceived competence, which could be a possible predictor of intrinsic motivation [41]. The physiological explanation of the association between having a good fitness level and higher motivation could be related to the release of endorphins during aerobic and resistance fitness, which promote feelings of well-being and positive emotions [42]. However, in our study, a direct association was found between cardiorespiratory fitness and motivation, but as this is a cross-sectional study, the direction of the association could not be determined. Finally, the fit group reported higher motivation. In this sense, achieving an acceptable fitness level could be related to higher motivation during physical education lessons. Increased motivation during physical education should be related to higher physical activity during leisure time and the promotion of healthy behaviors [43].

The association between cardiorespiratory fitness and motivation was stronger when music was used. A more positive emotional state was related to the use of music during physical activity, where it enabled one to dissociate from internal sensory signals and focus on other factors [44], thus making it a more pleasurable experience than when under normal circumstances [45]. This increased pleasurable experience that music induces could be related to the higher levels of performance and perceived enjoyment associated to music in interval exercises [23]. This is partially in agreement with a recent study in adolescents, which showed that listening to motivational music was associated with a significant improvement of cardiorespiratory fitness in the treadmill run, but not in the SRT-original [46]. Accordingly, music could contribute to prolonged exercise durations at higher intensities [47]. Even more, music could be related to all the emotional systems [48] which have been related to motivation [49].

The results of this study are very important to researchers and practitioners. The relation between motivation and physical education has been studied, with girls being less motivated than boys [50,51]. Accordingly, increasing girls’ motivation levels should be crucial to increase the positive effects and adherence of exercise. The use of music, in accordance to the results of our study, could increase the percentage of girls who meet the minimum fitness level to be considered fit. In addition, music has been shown to strengthen the association between cardiorespiratory fitness and motivation. In this sense, the use of music in endurance activities with students should be a key for physical education teachers to increase motivation along with cardiorespiratory fitness. Further studies are needed in order to examine the role of physical activity on the associations between cardiorespiratory fitness and motivation in adolescents.

This study presents some limitations that are hereby to be mentioned. The same students who developed the two tests (SRT-original and SRT-music) and the sample selection process were not randomized and were from one city, which may have compromised the generalization of the results. In addition, the study was cross-sectional and did not allow us to establish the direction of the associations. Additionally, the lack of inclusion of physical activity data, as a confounder, could be considered as another limitation. However, this study also presents several strengths, such as the big sample size and the selection of feasible and valid tests to assess cardiorespiratory fitness and motivation levels.

## 5. Conclusions

Boys presented with higher levels of cardiorespiratory fitness and motivation than girls. In addition, cardiorespiratory fitness was associated with higher motivation, and this association was stronger when music was used in the SRT test. In addition, the fit group also reported a higher level of motivation towards physical education. In this sense, including music in activities focused on cardiorespiratory fitness could increase cardiorespiratory fitness performance and motivation, especially in girls. It should thus be important to increase adolescents’ cardiorespiratory fitness in order to increase motivation in physical education lessons and to include more motivational activities in order to achieve higher levels of performance.

## Figures and Tables

**Table 1:** Descriptive data of the participants.

Characteristics	Total	Boys	Girls	*p*
	*n* = 341	*n* = 163	*n* = 178	
Age (years)	14.24 (1.48)	14.26 (1.54)	14.22 (1.43)	0.825
Body mass index (kg/m^2^)	21.98 (4.34)	21.79 (4.71)	22.15 (3.97)	0.454
Weight status (*n* %) *				
Normal weight	200 (65.4)	97 (64.7)	103 (66.0)	0.548
Overweight	75 (24.5)	35 (23.3)	40 (25.6)	
Obesity	31 (10.1)	18 (12.0)	13 (8.3)	
SRT-original				
Cardiorespiratory Fitness (stages)	5.70 (2.74)	6.85 (2.90)	4.62 (2.07)	**<0.001**
VO_2_max estimated (ml/kg/min) †	43.66 (7.97)	46.78 (8.12)	40.73 (6.62)	**<0.001**
HRmax (beats/min) ‡	177.44 (29.44)	182.83 (26.2)	172.39 (31.44)	**0.002**
Fitness category (*n* (%))				
Fit	238 (76.3)	108 (71.5)	130 (80.7)	0.056
Unfit	74 (23.7)	43 (28.5)	31 (19.3)	
SRT-music				
Cardiorespiratory Fitness (stages)	6.22 (2.71)	7.43 (2.81)	5.15 (2.10)	**<0.001**
VO_2_max estimated (ml/kg/min) †	45.02 (7.94)	48.35 (7.81)	42.08 (6.84)	**<0.001**
HRmax (beats/min) ‡	176 (31.20)	182.76 (26.70)	170.64 (33.64)	**0.001**
Fitness category (*n* (%))				
Fit	256 (80.5)	111 (74.5)	145 (85.8)	**0.011**
Unfit	62 (19.5)	38 (25.5)	24 (14.2)	
Motivation				
Self-perceived competence	3.23 (0.92)	3.50 (0.87)	2.98 (0.89)	**<0.001**
Compared competence	2.48 (1.06)	2.82 (1.01)	2.18 (1.02)	**<0.001**
Commitment	3.89 (0.69)	3.96 (0.68)	3.83 (0.68)	0.077
Anxiety	2.36 (0.97)	2.14 (0.88)	2.55 (1.03)	**<0.001**

* Classified according to the World Obesity Federation cut-offs [29]. † VO_2_max= maximum oxygen uptake estimated by Leger et al., Equation [9]. ‡ HRmax = maximum heart rate. The fit and unfit group was classified based on the Ruiz et al. [28] cut-off points for cardiorespiratory fitness. Statistically significant values are shown in bold.

**Table 2:** Associations between cardiorespiratory fitness and achievement motivation levels.

Fitness	Motivation			
Cardiorespiratory Fitness	Self-Perceived Competence	Compared Competence	Commitment	Anxiety
	β (95%CI)	β (95%CI)	β (95%CI)	β (95%CI)
SRT-original				
Cardiorespiratory fitness (stages)	0.36 (0.08, 0.16) **	0.34 (0.09, 0.18) **	0.18 (0.01, 0.07) *	−0.34 (−0.16, −0.08) **
VO_2_max * estimated (ml/kg/min)	0.39 (0.03. 0.06) **	0.37 (0.03, 0.07) **	0.19 (0.00, 0.03) *	−0.37 (−0.06, −0.03) **
HRmax ** (beats/min)	0.01 (−0.00, 0.00)	0.03 (−0.00, 0.00)	0.06 (−0.00, 0.00)	0.08 (−0.00, 0.00)
SRT-music				
Cardiorespiratory fitness (stages)	0.43 (0.11, 0.19) **	0.41 (0.11, 0.21) **	0.24 (0.03, 0.09) **	−0.38 (−0.18, −0.09) **
VO_2_max * estimated (ml/kg/min)	0.46 (0.04, 0.69) **	0.45 (0.04, 0.08) **	0.26 (0.01, 0.03) **	−0.41 (−0.07, −0.03) **
HRmax ** (beats/min)	−0.02 (−0.00, 0.00)	0.05 (−0.00, 0.00)	0.05 (−0.00, 0.00)	0.13 (0.00, 0.1) *

β = standardized coefficient. Statistically significant values are shown in bold. * *p* < 0.05. ** *p* < 0.001.
